# A Systematic Treatise, Etc., on the Principal Diseases of the Interior Valley of North America, as They Appear in the Caucassian, African, and Esquimaux Varieties of Its Population

**Published:** 1850-07

**Authors:** 


ARTICLE VI.

*A Systemactic Treatise, Historical, Etiological, and Practical, on the Principal Diseases of the Interior Valley of North America, as they appear in the Caucassian, African, Indian, and Esquimaux Varieties of its Population*: By Daniel Drake, M. D. Cincinnati; Winthrop B. Smith & Co. Philaeelphla; Gregg, Elliott & Co. New York; Mason & Law, 1850; pp. 878 oct. (From the publishers, and for sale by A. H. & C. Burley, Chicago.)

A book is here presented. worthy of the name; one which has cost as much labor to produce it as any medical work yet published by an American author—one that presents a greater amount of Hydrographical, Meteorological Physiogical, and Social information in reference to the etiology of the Interior Valley of North America than is to be found collated in reference to any other country so far as we are informed—one that is made up of an original collection of important facts which render the work equal in value to any American medical publication that has yet gone to press, and which will give it as enduring a reputation as can reasonably be claimed for any other—a book that is highly pleasing in style, and yet free from the appearance of a set effort to be entertaining. It interests because in almost every page it communicates to the physician of the country of which it treats, such information as he desires to have.

We feel proud for the West, that such a work has not only been produced but also published here, in a style of execution that will compare favorably with the works of the most celebrated publishing houses in the country.

To give at the same time the plan of the work before us, and to show the high estimation of the work by an esteemed trans-Atlantic contemporary, we give the following from the Medical Examiner for June :

" Book first treats of *General Etiology*, which is discussed under the heads or parts of 1. *Topographical and Hydrographical Etiology ;* and 2. *Climatic Etiology*. The second, which is the most attractive theme, cannot however, be either studied or understood to any extent, without the aid of the first; and the two jointly are of a paramount value, we might say necessity, for a clear knowledge of the diseases described in a subsequent part of the work. It would be a grave mistake in the reader and injustice to the author, not to see a unity of plan and purpose in its numerous divisions. Many of these, which, at first sight, would seem to be matters of allowable philosophical curiosity, merely, or at most, collateral or even introductory, are, truly, an integral and indispensable part of the treatise ; and they could not be detached from it without marring its proportions, if not actually destroying its characteristic and atttractive features.

The common constituents of climate, in temperature, atmospheric pressure, and dryness and moisture, force and direction of the winds, and electricity, are greatly modified, independently of latitude and the succession of seasons, by the localities, whose peculiarities are included under the head of *Topographical and Hydrographical Etiology.* This is first exhibited in a "*general analysis*," and then in connection with successive portions of the "*southern Hydrographical Basin*," which includes the Gulf of Mexico and the different towns and harbors and inlets from Vera Cruz to Mobile, and the *Delta of the Mississippi.* Thence, in one direction, we follow along the bottoms and bluffs of this river on to the Upper Mississippi, and, in another line, the regions "*West of the Gulf and of the Mississippi River.*" In fine, the entire regions on each side of the "Father of Waters," up to the Ohio Basin and the countries north of this latter, are spread before us by the author, who has thus collected and concentrated an amount of medical hydrography and topography of the great valley of the Mississippi, hitherto either unknown, or to most of us unattainable.

Next we are taken over the "*Eastern or St. Lawrence Basin*," including the regions of the lakes and of the river itself; and the first part is concluded by a sketch of the "*Hudson and Arctic Hydrographical Basins.*"

Part second is devoted to "*Climatic Etiology*," under its appropriate divisions. Part third furnishes us with "*Physiological and Social Etiology*," especially of the Caucassian portion of the Great Valley, in their physiological characteristics, diet and use of stimulating and narcotic substances; also in their clothing, lodging, bathing and habitations, and in their occupations, pursuits, exercise and recreations. The Hippocratic advice given at the beginning of this notice, has been fully followed out by Dr. Drake; and no physician can henceforward pretend to practice medicine in the great valley of North America, without having first possessed himself of the Systematic Treatise and studied carefully its contents, that so he may prepare himself for a suitable treatment of the diseases in his town or district, and for filling up from his own observation the great outlines furnished in this work.

Book second exhibits the application of all that preceded it to a review of the geographical and topographical; in fine, the climatic causes of Febrile Diseases; beginning with Autumnal Fever, being that which has the widest range, and attacks the greatest number of people. The divisions of this latter, described by Dr. Drake, are, first, Intermittent Fever, with its varieties of simple, inflammatory and maligant, and then Remittent Fever, considered as simple and malignant. The last two chapters are given to a consideration of the "*Pathological Anatomy and consequeuces of Autumnal Fever.*"

From the preceding outline our readers may acquire some idea of the abundance and variety of the topics examined in the present volume. Of their richness and intrinsic worth, they can only obtain accurate notions by reference to its pages. Even if more space were allotted to us than is possible within the assigned limits of this journal, we should still fail to do justice to the multiplied and pains-taking researches, statistical and other tabular estimates, as of temperature, barometrial changes and seasons, interspersed with judicious summaries and apposite statements of the material facts, with which this volume abounds."

To give a review of the work that should do justice to it would carry us entirely beyond the limits prescribed for this department in the present number. In fact, we do do not see how a review, short of occupying an entire number of our Journal, could give anything like a satisfactory view of the contents of the volume. We take leave of it for the present, hoping to have space hereafter to devote to it; in the mean time assuring our readers that the better plan will be for each of them to procure and carefully study it for themselves. E.

